# Vulnerability of cities to toxic airborne releases is written in their topology

**DOI:** 10.1038/s41598-021-02403-y

**Published:** 2021-11-29

**Authors:** Sofia Fellini, Pietro Salizzoni, Luca Ridolfi

**Affiliations:** 1 Laboratoire de Mécanique des Fluides et d’Acoustique, UMR CNRS 5509, Université de Lyon, École Centrale de Lyon, INSA Lyon, Université Claude Bernard Lyon I, 69134 Écully, France; 2 Department of Environmental, Land and Infrastructure Engineering, Politecnico di Torino, 10129 Turin, Italy

**Keywords:** Environmental sciences, Fluid dynamics, Complex networks, Urban ecology

## Abstract

The incidental or malicious release of toxic gases in the atmosphere is one of the most critical scenarios for cities. The impact of these releases varies with the ventilation potential of the urban environment. To disentangle this crucial aspect, vulnerability to airborne releases is here traced back to essential properties of the urban fabric. To this aim, pollutant dispersion is disassembled in its fundamental bricks and the main drivers of the process are captured. The analysis is based on four cities with emblematic architectures: Paris, Firenze, Lyon and New York. Results show that vulnerability is driven by the topology of the city and by its interaction with the approaching wind. In this sense, fragility to toxic releases is written in the layout of the urban fabric and results from its historical evolution. This study paves the way to the assessment of air pollution-related issues from a morphological point of view.

## Introduction

With two thirds of the world’s population expected to live in cities by 2050^1^, the transition to sustainable and livable urban environments is the key challenge for urban practitioners and policy-makers. In recent years, the scientific community has therefore been questioning what are the best models of sustainable cities^2^, the best policies for urban health^3^ and urban shape^4^, and the challenges given by human–environment interaction^5^. In this framework, the management of air quality plays a crucial role, as in urban areas large populations are exposed to a large variety of persistent pollutant sources in a poorly-ventilated environment, as reviewed in^6–8^. According to the World Health Organization (WHO)^9^, air pollution causes around seven million premature deaths each year. Due to their population density and low ventilation potential, urban areas are also particularly vulnerable with respect to accidental releases such as those related to gas leaks, industrial plants or the transport of hazardous material. Evidently, this has also a clear link to city security^10^, since toxic substances could be maliciously dispersed in the urban atmosphere for terroristic purposes.

To address these issues, many efforts have been invested by the scientific community to develop accurate models of pollutant dispersion in urban areas. The main modelling methodologies are discussed in^11,12^. Generally, the focus is on simulating the dynamics of a turbulent flow around buildings, and how these drive the dispersion of airborne pollutants. The urban canopy is therefore represented as a set of -more or less detailed- obstacles invested by the wind blowing over the city. However, flow patterns within the urban canopy are strongly driven by the layout of buildings and the way street canyons cross each other, as revised in^13,14^. In this sense, the city structure plays an active role in conveying pollutants, especially for the transport dynamics at the pedestrian level. Consequently, the same pollutant source may have a different impact if released in cities with different urban plans or in different places within a same urban district.

In recent years, the link between urban morphology and air pollution has been investigated by focusing on key but single properties as the urban shape^15^, the packing density of buildings^16,17^ and the geometric characteristics of the street canyons^18^. However, a comprehensive analysis of how propagation of pollutants in the streets depends on the characteristics of the urban fabric is missing so far. Moreover, the way street connectivity and layout can affect urban ventilation is still an uncharted territory. In this work, we address this gap with the aim to shed light on one of the most challenging questions in the field of urban safety and planning: what makes a city or an urban area vulnerable to localized airborne releases? How much does topology, in which the history of a city is written, affect ventilation in the streets and thus the vulnerability of the city in case of a toxic point source?

To answer these questions we first outline the concept of vulnerability, as introduced in^19,20^ and reported in the Methods section. Considering a gaseous release in a specific place within the urban canopy, we define the vulnerability of this place as the extent of the area contaminated by the localized release. The number of people exposed to the contamination is not taken into consideration to prevent the vulnerability analysis from being overwhelmed by the intrinsic temporal variability and unpredictability of citizens’ distribution. To estimate the vulnerability of each location in the urban tissue, we adopt an innovative model that we developed by exploiting tools from complex network theory. The application of a complex network approach in urban science is in line with recent applications as those proposed by Crucitti et al.^21^ who have adopted metrics from complex network theory to analyse the structural properties of urban areas, Barthelemy^22^ who applied concepts from statistical physics to understand the structure and dynamics of cities, and Zischg et al.^23^ who used complex network analysis to investigate the topological coevolution of urban infrastructures.

In our model, the urban canopy is represented as a network where links and nodes are the streets and street intersections, the direction of the network links follows the direction of the wind along each street, while the physics of pollutant dispersion is enclosed in a fluid dynamic weight for the network links (see Eq. 4 in “Methods”). In this complex network representation, local vulnerability is easily computed (in a variety of meteorological conditions) by means of a centrality metric^20^ that associates to each node in the network a value based on its spreading potential (Eq. 5), and thus on the extent of the contaminated area when a release takes place in the node.

Complex network-based modelling offers three key features for our purposes: (i) compared to Computational Fluid Dynamics (CFD) simulations, vulnerability can be assessed very quickly for each node of a city network, thus enabling detailed mapping of the impact of a localized release in every place in the city. A low computational cost is mandatory in this work as we, for the first time, analyze the vulnerability of different cities under multiple weather scenarios; (ii) model outcomes reliably describe pollutant dispersion (see “Methods”), in particular in the first urban blocks downwind the source where contamination in the streets is predominant compared to unconfined dispersion above roofs; finally, (iii) urban vulnerability to toxic releases is expressed analytically by a single physically-based metric containing all the essential information, both structural and dynamic, to describe the spreading potential of a place in the city. The last point is crucial for the aim of this work, since starting from this information-rich object (i.e., the centrality metric for urban vulnerability) and disassembling it, we are able to identify the key drivers of pollutant propagation in the streets. The decomposing process of vulnerability into evident urban quantities is the original focus of this work and brings out an unprecedented result: the dominant role of urban topology in the vulnerability of a city to localized air releases.

## Results

**Figure 1:**
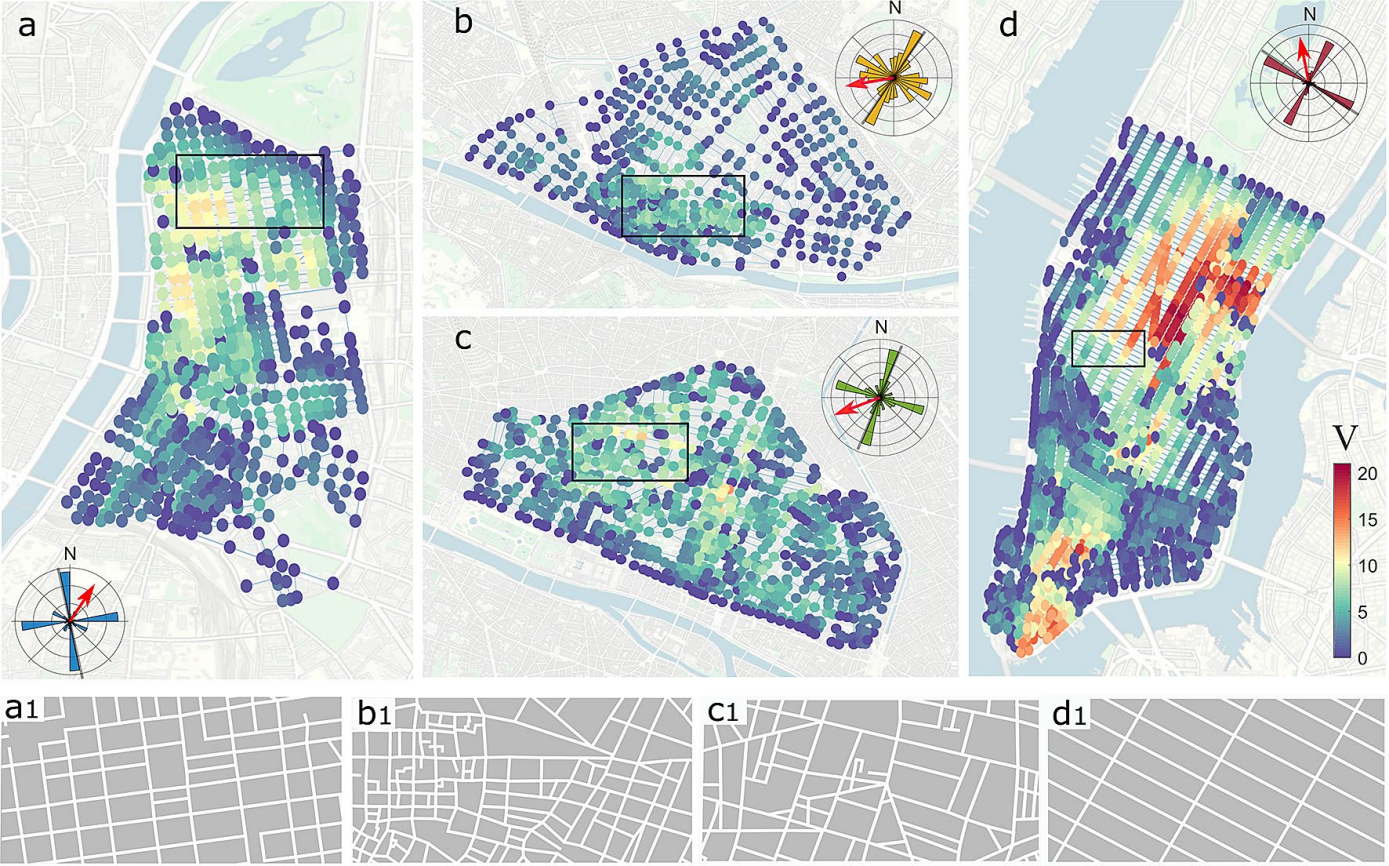
Vulnerability maps for **(a)** Lyon, **(b)** Firenze, **(c)** Paris, and **(d)** New York for a wind direction of $45^\circ$ with respect to the main axis of the urban fabric. The polar histograms in the insets report the distribution of street orientation, while the red arrows represent the wind direction with respect to the street network. Panels **a1**–**d1** show the urban pattern in a rectangular area of 0.5 km$^2$ (reported in panels **a**–**d**) for the cities of Lyon, Firenze, Paris, and New York, respectively. Background images made with QGIS 2.18 (https://qgis.org).

As a starting point, we compare the vulnerability of four districts in Lyon, Paris (France), Firenze (Italy) and New York (US). These cities were chosen as emblematic of different topologies, resulting from different historical urban layering. The historic center of Firenze (panel b in Fig. 1) is mainly characterized by a dense urban fabric with a medieval signature of narrow and winding streets^24^. In Paris (panel c), Haussman’s renovation plan at the end of the 19th century supplemented the North–South and East–West ancient crossroad by a second network of concentric large avenues^25^. The rectilinear grid of Manhattan, New York, originates from 1811^26,27^ and extends along the spine of Manhattan island (panel d). Despite the significant difference in size, a similar regular pattern is found in the modern urban area of Lyon (panel a), developed in the second half of the 19th century. In the insets of Fig. 1, we report for each city a polar histogram of the orientation of the streets. Although greater variability is observable for the orientation of the streets in the urban areas of Firenze (panel b) and Paris (panel c), two main orthogonal axes are found in the spatial structure of each city.

The urban networks analysed in this work were delimited in order to be large enough to include the distinctive patterns of these four cities. The edges of the areas were traced along physical boundaries (e.g., rivers, parks, railways, large avenues) which act as elements of discontinuity in the dispersion process. Where not possible, the break was forced along wide streets.

We promptly computed vulnerability maps for the selected urban areas by means of the centrality metric we derived in^20^ and recall in the “Methods” section. The nodes with the highest centrality values (*V*) are the most vulnerable as they correspond to the best spreading locations in the urban fabric. The spreading potential of a node is evaluated based on the extent of the area that is contaminated when the release takes place in this same node.

We report in Fig. 1 the vulnerability maps of the four urban areas for the indicative scenario of a wind blowing at an angle $\phi =45^\circ$. In the insets of Fig. 1, the wind direction is indicated with a red arrow. Given the different orientation and structure of the street networks, $\phi$ is defined as a clockwise angle with respect to the main axis of the city, which is identified as the longest bar in the polar histogram of street orientation.

**Figure 2:**
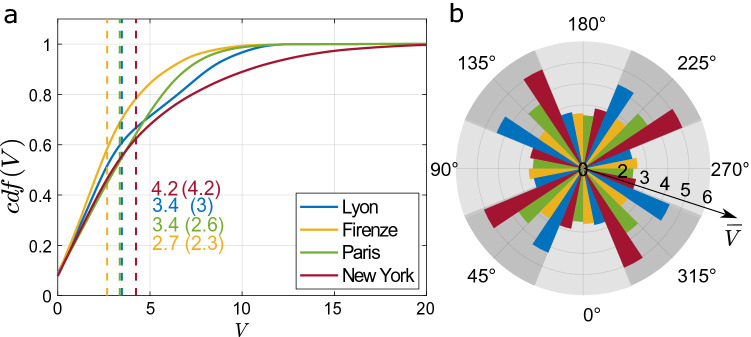
Vulnerability distribution for different cities and wind directions. **(a)** *Cdf* of node vulnerability for the different cities under eight different wind directions. The mean vulnerability is shown as a dashed line and reported numerically together with the standard deviation (in parentheses). **(b)** Mean vulnerability of city networks for each wind direction. Colors blue, yellow, green and magenta correspond to the urban networks of Lyon, Firenze, Paris and New York, respectively.

To extend the analysis to multiple meteorological scenarios, we estimated the vulnerability of each node (seen as a spreading source) for eight different wind directions ($\phi =0^\circ$, $45^\circ$, $90^\circ$, $135^\circ$, $180^\circ$, $225^\circ$, $270^\circ$, $315^\circ$). In this way, for each city, we obtained an extended dataset of vulnerability values that we represent in a compact way by means of a cumulative distribution function, as shown in Fig. 2a. The intercept of the *cdf* represents the nodes with null vulnerability. These are mostly located along the physical edges of the domain where the pollutant gas is blown away by the wind without affecting other streets. Where the delimitation of the network is forced (for example on the sides of central park as regards Manhattan), the interruption of the propagation, in the vulnerability model, is also constrained. This does not result in any artificial effect when the boundary is located upwind with respect to the network (propagation carries on from the boundary towards the considered urban area). On the other hand, when the boundary is downwind, vulnerability can be there underestimated. Considering the multiple wind directions simulated and the small number of nodes belonging to these edges (1% of the total number of network nodes), this effect has been calculated negligible to the purposes of this work.

According to the mean values (vertical dashed lines) of the distributions reported in Fig. 2, New York is the most vulnerable city on average, while Firenze is the most protected. The vulnerability of New York and Lyon are the most sensitive to changes in wind direction, as shown by Fig. 2.b, where a polar histogram reports the mean vulnerability for each city for the eight directions of the approaching wind. In general, the spreading potential is more effective when the wind is oblique ($\phi =45^\circ ,$ $135^\circ$, $225^\circ$, and $315^\circ$) to the main orthogonal axes of the street network, as evidenced by the higher vulnerability observed for the dark gray sectors of Fig. 2b. We also notice that vulnerability for parallel ($\phi =0^\circ ,$ $\phi =180^\circ$) and perpendicular ($\phi =90^\circ ,$ $\phi =270^\circ$) wind directions is quite similar. This seems counterintuitive as previous studies (e.g.,^28,29^) have reported that a perpendicular wind is much more unfavorable for the dispersion of pollutants in a street. In this regard, we underline that $\phi$ is here defined with respect to the main axis of the city, so for $\phi =90^\circ$ not all streets will be perpendicular to the wind direction. For example, in the regular network of Manhattan we expect the number of perpendicular streets to be similar to that of parallel streets, when $\phi =90^\circ$.

The reasons for the different resilience of cities (and their patterning) to gas propagation are embedded in the centrality metric adopted to compute urban vulnerability. The key factors for node vulnerability can then be analytically recognized in the metric definition (Eqs. 4–5 in “Methods”): the highest vulnerabilities are achieved when the set of reachable nodes ($\mathcal {V}$) from the source node is large, and the paths connecting the source and the reachable nodes ($d_{sr}$) are short, i.e. the propagation cost ($\omega$) along the paths is minimal. In other words, the spots in a city (i.e. nodes in a network) with the highest spreading potential are those from which a toxic plume can reach many other locations with significant concentration. Going beyond the vulnerability results, we aim here to decompose the aforementioned elaborate and meaningful quantities (the set of reachable nodes, the shortest paths, the propagation cost) in elementary properties of the urban area in order to link the vulnerability of a city to its tangible characteristics.

We start by disassembling the propagation cost associated to each street. Given a source node, a pollutant plume will propagate along the streets downwind the node. The propagation cost of each street (Eq. 4) describes the decay of concentration that the plume undergoes when it propagates along the street. Neglecting physico-chemical transformations, this cost depends on the transport processes within the streets and is a function of two dimensionless quantities: a geometrical ratio between the length (*l*) and height (*h*) of the street canyon, and a dynamic ratio between the exchange rate of pollutants towards the atmosphere above roof level (*v*) and the advective velocity along the longitudinal (*u*) axis of the street. According to^30^ and^31^, these two velocities can be parametrized as a function of the external wind intensity, the cosine ($\theta$) of the angle between the wind direction and the orientation of the street, the geometry of the street canyon (its length *l*, height *h* and width *w*) and the aerodynamic roughness of building walls. As detailed in the Methods, the dependence of the propagation cost on the external wind intensity disappears as both velocities *u* and *v* scale linearly with it. Assuming constant aerodynamic resistance of the surfaces, the parameters *l*, *h*, *w*, $\theta$, remain the relevant building blocks for the propagation cost along a street.

We underline that the parametrizations adopted here for the transport mechanisms in a street are based on the up-to-date literature and are currently employed in operational models (see the Methods section for mode details). Any refinements to this transport model may be included in the future. In this case, the cost associated to each street may depend on additional parameters that, however, we expect to be of second-order importance to those listed above.

While pollutant transport in a single street canyon (i.e. the propagation cost) has been easily broken down into its basic elements, the information enclosed in the shortest paths ($d_{sr}$) and in the set of reachable nodes from the source ($\mathcal {V}$) is much more challenging to trace back to evident properties of the city. These quantities depend on the sequence of streets that must be traveled to connect a source node to the surrounding nodes, i.e. on the way the streets are interconnected. The information is thus primarily topological. However, we point out that the interconnectivity of the network is not frozen, but dynamic, as it is given by the reaction of the urban structure to the direction of the external wind. In fact, the links of the street network are directed according to the orientation of the approaching wind. Moreover, the connectivity between the nodes is limited by the decay of the concentration along the streets. Although a target node may be reached from the source node by means of a path across the network, the two nodes may not actually be connected by a propagation path as the pollutant concentration may vanish along the path. For these reasons, traditional descriptors of network topology cannot be applied directly to describe the topological component of the vulnerability. Instead, we have to look for tailored and simple indicators that can express the wind-driven interconnectivity of the street network and the reachability potential between the nodes.

Focusing on a node as spreading source, we infer that the number of links in its downwind area gives a first estimate of the potential for a release in the node to affect many other locations in the network. To delimit this downwind area, we adopt the concept of *n-hop* neighborhood^32,33^. Two nodes are *n* hops apart if it is possible to reach the target node from the source node by traveling *n* links. We identify the downwind area of the source node as the subnetwork composed by the nodes that are reachable from the source via at most *n* hops along the directed links. We propose the number of links in this neighborhood (*k*) as a suitable measure of reachability from the node. This reachability depends upon three features: (i) the local structure of the street network, (ii) the direction of the wind, and (iii) the topological distance *n*. This latter parameter is intuitively correlated to the intensity of the release. More precisely, it depends on the ratio between the magnitude of the toxic release at the source and the threshold value for pollutant concentration to be significant. In this work, *n* is taken as constant and its value is obtained from an optimization analysis detailed in the “Methods” section (Fig. 6) .

Once the $n-hop$ neighborhood of a node is delimited, the number of links *k* is not exhaustive in giving information about the properties of the paths connecting the source to the other nodes of the neighborhood. For the same *k*, different structures of the neighborhood can take place (see Fig. 6b), with consequent different outcomes for the propagation process that we are breaking down to basic components. The higher the number of links outgoing each node of the neighborhood, the higher the potential concentration for the *k* links, as they are topologically closer to the source. This feature can be accounted for by means of a simple branching index (*b*) for the node neighborhood, defined in Eq. 8 as the average outdegree for the nodes belonging to the neighborhood^34^.

The disassembling analysis presented above suggests that the spreading potential of a node, and thus its vulnerability, mainly depends on the topological parameters *k* and *b* and on the geometrical characteristics of its neighborhood, i.e. *L*, *H*, *W*, $\Theta$, where the capital letters are used to indicate the local average (over the *n-hop* neighborhood) for the length (*l*), height (*h*), width (*w*), and orientation ($\theta$) of the street canyons.

In adopting averaged geometrical properties, we are assuming that these characteristics are rather homogeneous in the surroundings of a node. While the height, width and length of the street canyons are actually quite uniform on a local scale, especially in European city centers, the same does not apply to the orientation of the streets. The streets of a neighborhood intersect each other at different angles (e.g., at $90^\circ$ in grid plans), and the intensity of the wind in the streets changes strongly with their orientation. Low wind streets act as bottlenecks in the propagation paths, thus strongly influencing the spreading dynamics. For this reason, the standard deviation of street orientation in the neighborhood ($\sigma$) is expected to be an additional topological index of node vulnerability.

To assess whether the identified parameters are valuable basic elements of node vulnerability, we perform a regression analysis adopting a simple (but versatile) non linear model of the form:

1 $$\begin{aligned} V_{pred}=\alpha L^\beta H^\gamma W^\delta \Theta ^\epsilon k^\zeta b^\eta (1-\sigma )^\lambda . \end{aligned}$$

We estimate the coefficients $\alpha$ to $\lambda$ by means of a nonlinear least square technique (namely the *fitnlm* function in Matlab) that minimizes the sum of the squares of the residuals between the predicted vulnerability $V_{pred}$ and the vulnerability *V* obtained from the centrality metric (Eq. 5 in “Methods”). The regression is performed considering all the scenarios presented in this study: four different urban networks and eight different wind directions. The *p*-values for the coefficients $\alpha$ to $\lambda$ tend to zero, indicating that the relationships between the independent variables and the observations (*V*) are statistically significant. Note that in Eq. (1) we adopt $1-\sigma$ as predictor, instead of $\sigma$, to avoid null entries, as $\sigma$ takes value in [0 1). To explain the reason for this range for $\sigma$, we point out that the angle between the wind direction and the street axis is defined in [$-90^\circ$ $90^\circ$]. As a consequence, the cosine ($\theta$) of the angle varies in [0 1] and the standard deviation of $\theta$ (i.e. $\sigma$) varies in [0 1).

**Figure 3:**
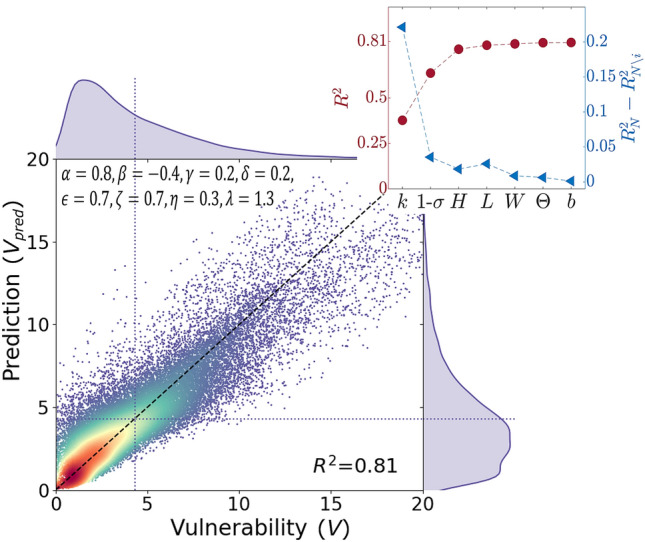
Correlation of node vulnerability with basic geometrical and topological parameters of the street network. Color (blue to red) is associated to point density. Left *y-axis of inset*: trend of the coefficient of determination $R^2$ as the urban indicators are progressively included in the model. Right *y-axis of inset*: unique contribution of the indicators.

The scatter plot in Fig. 3 compares $V_{pred}$ against *V*. Points correspond to the nodes of the four urban networks in the eight wind scenarios. The figure suggests that 80% of the spreading capacity (*V*) of a spot in a city can be grasped from the basic geometrical and topological characteristics of its neighborhood. To identify the most influential parameters in the regression, we evaluate the gain in the coefficient of determination $R^2$ as they are progressively included in the model (red circles in the inset). The quantities are entered in order to optimize $R^2$ at each addition. Alternatively, the role of each parameter can be evaluated adopting the concept of unique contribution (triangles in the inset), i.e. the loss in the coefficient of determination induced by the exclusion of the parameter from the model^35^. Both analyses reveal *k* and $\sigma$ as the main indicators for the vulnerability of a node. Actually, more than 60% of the total variance (inset in Fig. 3) is explained by these two parameters, unveiling the crucial role of topology in governing the dynamics of pollutants in urban areas. The effect of the geometrical properties (*L*, *H*, *W*, $\Theta$) of the street canyons is secondary. Among these, the contribution of the building height (*H*) is the most remarkable as its contribution, combined with that of the two topological parameters *k* and $\sigma$, brings the correlation to almost its maximum value.

Given these results, it is enlightening to show some tangible examples of how the three simple indicators *k*, $\sigma$ and *H* dominate urban vulnerability. We wonder which of these properties determine the distinct vulnerability of neighboring areas belonging to the same district, and which ones differentiate the resilience of cities with a different urban history.

**Figure 4:**
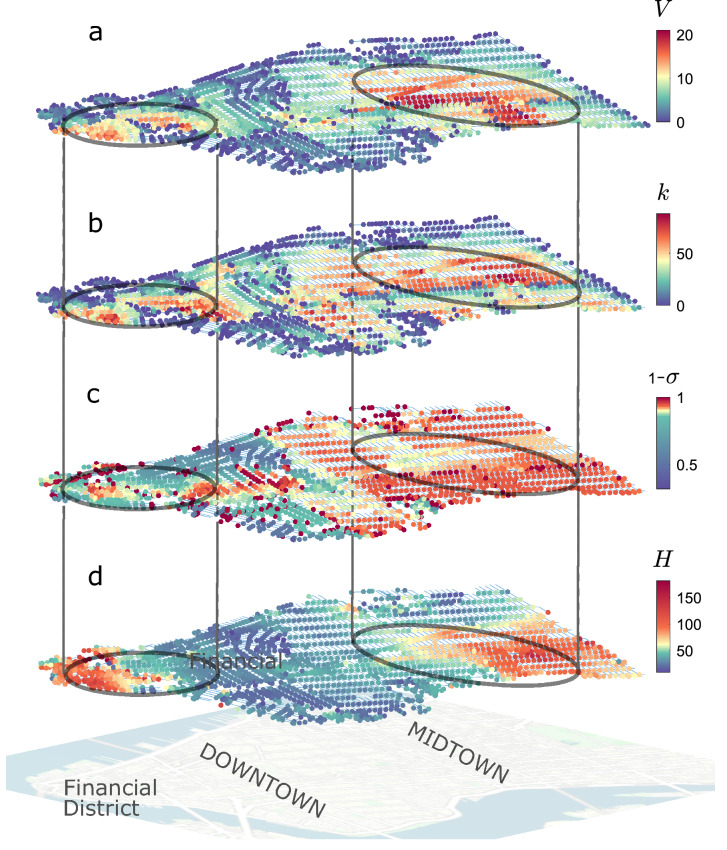
Street network of Midtown and Downtown Manhattan. Node color is associated to node vulnerability (*V*), and to its key indicators: street reachability (*k*), inhomogeneity in street orientation ($\sigma$), and average height of buildings in the node neighborhood (*H*).

Figure 4a shows the spatial distribution of the key parameters *k*, $\sigma$ and *H* and of node vulnerability, for Manhattan and a wind direction $\phi =45^\circ$. In panel b, high street reachability (*k*) is observed in the central part of Midtown, in the heart of Downtown, and near Wall Street. An homogeneous distribution in the orientation of the streets with respect to the incident wind (low values for $\sigma$ and thus high $1-\sigma$) is especially found in Midtown (panel c). Finally, in panel d, high-buildings (*H*) distinguish the Financial District and East Midtown. A perfect match between the four layers is not expected as vulnerability is given by the synergistic contribution of the different parameters. However, in line with the results of the regression model shown in Fig. 3, a positive correlation is observable between the most vulnerable areas (circled areas comprising the nodes with highest *V* in panel a of Fig. 4) and those with the highest values for the three indicators. In these areas, high buildings inhibit the vertical exchange of pollutants between the streets and the atmosphere above, as largely discussed in literature (see e.g.^36,37^). This inhibition limits the concentration decay along the propagation paths and facilitates large-scale contamination. Moreover, the great number (*k*) of streets topologically close to the node increase the impact of the release. The effect of $\sigma$ is significant especially for the vulnerability of Midtown. Here, since $\phi =45^\circ$ and the street network is regular, $\theta$ (the cosine of the wind-street angle) is almost the same for all the streets. Therefore, the standard deviation of $\theta$ ($\sigma$) is low and the predictor $1-\sigma$ is high. Physically, this means that the external wind approaches all the streets with almost the same angle. As a consequence, the intensity of the longitudinal wind in the streets is similar (the street aspect ratio is also similar) and the propagation takes place equally along both the dominant and lateral segments of the street network^38^, thus favoring the spread over large areas. Although high values of *H* and $1-\sigma$ can be detected in the North-East corner of Midtown too, here the vulnerability is mitigated by a higher discontinuity in the urban pattern (low *k*). This feature, together with the great overlapping of red areas in panel a with those in panel b, evidences the key role of street reachability (*k*) in the heterogeneity of vulnerability between areas of the same urban district.

**Figure 5:**
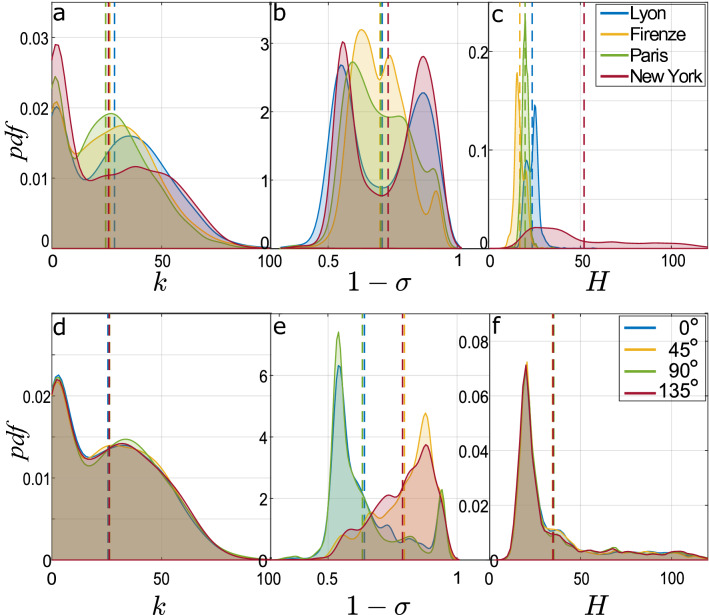
Probability density function of the key parameters *k*, $1-\sigma$, *H*. In the first row, each curve refers to a city and includes vulnerability data from eight different wind directions. In the second row, each curve corresponds to a specific wind direction and includes vulnerability data from the four cities, together.

From these observations, we move to a broader view and investigate the structural fragility of a city as a whole. In Fig. 5a–c, we report the probability density function (*pdf*) of the key parameters *k*, $1-\sigma$ and *H*. For each city, the *pdf* is calculated over all the network nodes and for the different wind directions. So, each *pdf* is representative of eight different networks for the same urban area. In panel a, the distributions for the four cities are quite similar but the tails of the *pdfs* highlight that the highest values for street reachability (*k*) occur in the street networks of Lyon and New York. The homogeneity in street orientation with respect to the wind (panel b), expressed by $1-\sigma$, exhibits a bimodal distribution and a slightly higher mean for the regular street network of New York. The two peaks are associated to distinctive wind scenarios, as will be discussed below. Also in this case, the observation of the tails of the *pdfs* reveals that high values of the vulnerability indicator are more probable in Lyon and New York. Finally, the distribution of building height (panel c) presents the most marked difference between the considered architectures, with high-rise buildings contributing to the heavy *pdf* tail of Manhattan. Comparing these results with those in Fig. 2a, Manhattan’s greatest vulnerability appears to be due to the greater depth of the urban canyons (high *H*) and the greater homogeneity, on average, in wind-street orientation (high $1-\sigma$). Conversely, the medieval structure of Firenze, with higher heterogeneity in street orientation (low $1-\sigma$) and low buildings (low *H*), enhances street ventilation and hinders propagation over long distances. Moreover, the tails of the *pdfs* for *k* and $1-\sigma$ reveal the role of topology in the higher variability of vulnerability values (given by the standard deviation of the *pdfs* in Fig. 2) for the street networks of Lyon and Manhattan.

After discussing the behavior of the single parameters, we assess the synergistic contribution of the three quantities. To this aim, we define a simple correlation index $\rho =\widehat{k} \cdot (1-\widehat{\sigma }) \cdot \widehat{H}$, where the hat denotes a *min-max normalization* of the parameters, i.e. the range of values of each parameter is rescaled in [0, 1]. For the urban areas of Manhattan, Lyon, Paris, and Firenze, $\rho$ gives 0.039, 0.017, 0.012, and 0.011, respectively. This ranking complies with the ranking inferable in Fig. 2 for the average vulnerability of the cities. This result confirms that vulnerability occurs when the three parameters are correlated, as already evidenced in Fig. 4.

To make the picture even more fascinating, it is worth noting that the role of topology, shown above as key, is dynamic as it varies according to the direction of the wind impacting the urban fabric. In panels d to f of Fig. 5, the *pdfs* of *k*, $1-\sigma$ and *H* are distinguished for four wind directions ($\phi =0^\circ$, $45^\circ$, $90^\circ$ and $135^\circ$). For each angle, the statistics are calculated over the examined cities, together. Although wind orientation alters the direction of the network links, and thus the delimitation of the *n-hop* neighboring area of each node, street reachability (panel d) and building height (panel f) remain statistically invariant for the different wind directions, suggesting a rather isotropic structure of the urban fabric. On the other hand, the variability in street orientation with respect to the wind (panel e) presents two distinctive trends for wind directions aligned with or oblique to the main axes of the street network. To explain this behavior, we refer to the simple case of a grid-like urban plan, like Manhattan’s plan. When $\phi =0^\circ$ or $90^\circ$, $\theta$ (the cosine of the angle between the street and the wind direction) mostly switches between 0 (for the streets aligned with the wind) and 1 (for the orthogonal streets), resulting in a high standard deviation over the neighborhood (low $1-\sigma$). When $\phi =45^\circ$ or $135^\circ$, instead, the incident angle $\theta$ mainly takes intermediate values, leading to higher values for $1-\sigma$. This distinctive behavior is clearly detectable in the two peaks that we have observed in panel b for the regular grid of Lyon and New York. The left peak of the bimodal distribution corresponds to the scenarios with aligned wind directions, while the right peak occurs for oblique wind directions over the city. A more irregular street pattern in Firenze and Paris adds random contributions to the way the wind approaches the street, thus altering this bimodal shape. Going back to panel e, the greater homogeneity in wind-street orientation (higher $1-\sigma$) for $\phi =45^\circ$ or $135^\circ$ gives insights into the higher vulnerability found for the scenarios with these wind directions in almost all cities (dark gray sectors in Fig. 2b). This result is confirmed by the correlation ($\rho$) between the three rescaled parameters ($\widehat{k}$, $1-\widehat{\sigma }$, $\widehat{H}$). The correlation $\rho$ is estimated separately for the different wind directions, but considering the nodes from the four urban areas together. For oblique wind directions, $\rho$ is about twice ($\rho =0.035$) the value found for the aligned wind directions ($\rho =0.018$).

## Discussion

In recent decades, the science of cities has proposed a new way of understanding cities as complex systems^22,39^. The structural components and the socio-economic dynamics of the urban system intertwine and are conditioned by the surrounding environment. Geometric patterns, scaling laws and non-linearities emerge from these interactions and can only be grasped by looking at the system as a whole.

Within this vision, we have proposed a new way of addressing air pollution-related issues in cities based on an overall analysis of the structural and dynamic properties of the urban canopy. While urban pollution models aim to simulate concentration in the streets with the highest possible resolution, the objective was here to unveil the key mechanisms underlying the ventilation of the urban environment. To this aim, we started by adopting an essential model able to assess the spreading potential of different places in the city. This model was applied to four urban areas characterized by distinctive topologies, sizes and properties of the buildings. The city networks were delimited in order to follow the physical edges of the urban areas. Where not possible the borders have been forced (this concerns around 1% of the total nodes). While this boundary effect may slightly alter the vulnerability results along these edges, it does not affect the analyzes reported in this work that are focused on investigating the link between the vulnerability of an urban node and the structural and architectural properties of its surroundings.

By decomposing the analytical model for urban vulnerability, we isolated the fundamental variables that drive the phenomenon and revealed the dominant role of urban topology. Two topological indicators of the area surrounding a node, *k* and $\sigma$, proved to account for more than 60% of the spreading potential. These two key quantities describe the reachability from the node across the directed network and the homogeneity in the orientation of the streets with respect to the wind direction. It follows that the impact of urban topology on vulnerability to airborne releases is not constant but depends on how the urban structure interacts with the approaching wind. Significant, though more intuitive, is the role of canyon geometry, with high buildings acting as a trap for pollutants.

These general findings pave the way for a number of applications and refinements. Once the fundamental role of topology (dynamically evolving with the external wind direction) has been captured, the characteristic vulnerability of a city can be estimated by taking into account the actual wind rose in the site. As shown in Fig. 5, this will define a distinctive distribution of $\sigma$. Variations in wind patterns due to climate change or urban transformations may affect the *pdf* of wind direction, and therefore that of $\sigma$, in the near future. In this sense, the distribution of the topological parameter $\sigma$ acts as an indicator for the susceptibility of the city’s vulnerability to climatic variations.

This observation highlights the advantages of having broken down vulnerability into basic elements attributable to well-defined urban characteristics. In this regard, we note that the indicators identified so far account for about 80% of the vulnerability. The application of the proposed method to a larger basket of cities may evidence anomalies and bring out new topological indicators that further improve the correlation.

Besides being versatile and suitable for further refinements, our method has the advantage of tracing a complex fluid dynamics phenomenon to evident characteristics of the urban fabric, which can be addressed directly by urban practitioners. Our findings clearly show that vulnerability is engraved in the topology of the city, and results from its history, evolution and urbanization process. On the basis of this intrinsic fragility, urban planners and risk managers can act to control, prevent and reduce risks related to airborne dispersion. Moreover, our study provides first-order guidelines to identify the most vulnerable areas in a city, to predict the consequences of interventions in the urban fabric, such as the opening or closing of dead-end streets, or to find the optimal location for sensors for real-time monitoring of the most critical places in the city.

## Methods

### Model for gas propagation in the streets

Flow and dispersion dynamics in the urban atmosphere are characterized by complex patterns induced by the presence of buildings (e.g., recirculating regions, wakes, channeling effects). Computational fluid dynamics (CFD) simulations^11,40^ can provide detailed information about these processes, as they solve the velocity and concentration field in the whole domain. However, when a district of hundreds of streets is considered, CFD simulations require computational costs that are not yet feasible for operational purposes. To balance computational speed and simulation accuracy, simplified modelling approaches have been therefore explored in the last decades^41^. These include street network models, as those proposed by^42–44^, that decompose the atmospheric domain in two sub-domains, the canopy layer and the atmosphere above. Above the canopy, the dispersion of pollutants is modelled using a Gaussian model suitably modified to take into account the anisotropy and inhomogeneity of the velocity field. In the canopy layer, instead, dispersion is simulated by parametrizing the transfers between neighboring streets and by solving a system of mass balance equations. Among street network models, SIRANE is currently used operationally in different cities in Europe to assess air pollution levels and citizens exposure to airborne contaminants. Recent applications are reported in^45–48^. SIRANE was validated against both wind tunnel experiments and field campaigns^49–53^, and has been shown to properly reproduce pollutant dispersion in urban areas. It is adopted in the Supplementary Information for a systematical validation of the complex network model we present below.

When the focus is on a single release, the computation of the concentration field in the entire urban domain is not necessary, as the contamination will mainly concern the area surrounding the source. From the perspective of the impact on citizens’ health and safety, the extension of the contaminated area is a preliminary measure of the vulnerability of the release location. In the framework of the development of operational models for emergency response, we proposed in^19,20^ a novel complex network approach to compute urban vulnerability to airborne releases. In the last decade, complex networks have demonstrated their great potential for the analysis of fluid-flow systems. Applications involve ocean transport^54–56^, turbulence^57,58^, and climate^59,60^. In the complex network perspective, we reformulate pollutant contamination in the urban atmosphere as a spreading process on a directed network. The links of the network represent the streets of the urban fabric, nodes are the intersections between streets, and the link direction is given by the direction of the longitudinal wind along the street canyon. Unlike urban dispersion models, the aim is not to reconstruct the concentration field in the streets but to evaluate the dispersion potential of each node in the network, i.e. the extension of the contaminated area from the node. In this way, node vulnerability can be assessed for multiple meteorological and release scenarios with a simplified model requiring low computational cost (2 orders of magnitude lower than SIRANE) and limited initial data.

The physical assumptions for pollutant dispersion in the streets are inspired by the model SIRANE, while the adoption of a complex network formalism is key to derive the analytical solution for node vulnerability.

The emission scenario is a point source releasing an inert gas at the pedestrian level, within a street canyon. Neglecting longitudinal turbulent diffusion and re-entrainment from the above atmosphere^19^, the transport along the street canyon can be simplified as follows:

2 $$\begin{aligned} \frac{\partial c}{\partial t}+u\frac{ \partial c}{\partial x}+\frac{v}{h} c=0, \end{aligned}$$

where *c*(*t*, *x*) is the concentration as a function of time *t* and space (longitudinal coordinate *x*).

The first two terms in Eq. (2) provide the advective transport driven by the mean velocity along *x*, referred to as *u*. This velocity is assumed to be given by a balance between the stress imposed at the canopy top by the external atmospheric flow and the drag due to the roughness of the canyon walls. Under these assumption, an analytical formulation for *u* was derived^30^ as a function of the external wind intensity (i.e. the friction velocity $u_*$) and direction, the geometry of the street canyon and the aerodynamic roughness of building walls. The third term in Eq. (2) models the vertical transfer between the street and the atmosphere above by means of a bulk exchange velocity *v*. This vertical transfer is both influenced by the external flow condition and by the properties of the street canyon, e.g., the canyon aspect ratio, the roughness of the building walls and the solar radiation^37^. As a first approximation, and as assumed by SIRANE, we adopt here the model proposed by Salizzoni et al.^31,61^, who verified experimentally that *v* scales on the friction velocity of the external flow ($u^*$). A similar model was also derived in^62^. The parametrizations for the transfer velocities (*u* and *v*) rely on a description of the atmospheric boundary layer in accordance with Monin-Obukhov’s theory, as customary in operational urban dispersion models (e.g., SIRANE, ADMS^42^). This modelling framework allows us to take into account the role of thermal fluxes between urban surfaces and the overlying boundary layer flow, i.e. those induced by solar radiation and the presence of other heat surfaces. This approach has two main limitations: on one side, as is the case for any other operational dispersion modelling approach (i.e. excluding computationally expensive numerical simulations over the all urban agglomeration), it is not suited to simulate air circulation for calm wind conditions. Secondly, the model does not take into account the effect of peculiar flow patterns within a street canyon. These are induced by differential wall heating, as enlightened by recent experimental investigations^37^. However, the adopted model for gas propagation in the streets is very ductile and the effect of other parameters (e.g., temperature of building walls, building geometry, presence of vegetation) could be easily included if new parameterizations for *u* and *v* become available.

In Eq. (2), the vertical transfer is the only decay term since it is assumed that the involved toxic substances do not undergo chemical or biological transformations or, in any case, have a reaction time significantly longer than the time needed for propagation. This hypothesis is in line with the objectives of the study, which focuses on the breathability of the urban area rather than on the dynamics of specific chemical species in the streets. However, a simple model for physico-chemical transformation could be easily implemented in the mass balance (Eq. 2) by adding a reaction term (e.g., $-kc$ in the case of a first-order decay^63^). Its effect would be embedded in the complex network model by changing accordingly the street weights that will be presented in the following section.

For both an instantaneous and a continuous release at the source, the solution^19^ for the concentration at the end of the street reads:

3 $$\begin{aligned} c(l/u,l) =c_0 e^{-\frac{l}{u} \frac{v}{h} }, \end{aligned}$$

where $c_0$ is the concentration at the source and *l* is the street length. Since both *u* and *v* are assumed in^30,31^ to be linear function of the friction velocity $u^*$ of the boundary layer flow, Eq. (3) results independent of the properties of the atmospheric boundary layer, such as the intensity of the external wind and the stability conditions.

In street intersections, the flow field is rather complex and with an intermittent behavior. Even slight variations in the building geometry and wind direction can affect the redistribution of pollutants from the upwind to the downwind streets, as already found in^64–66^. As a safety approach, we assume that the pollution front propagates towards all the streets downwind the intersection, keeping its concentration unaltered in crossing the intersection.

Based on these assumptions and adopting the network formalism, all the potential propagation paths from the source can be easily traced along the urban canopy. To this aim, a tree traversal algorithm on networks is efficiently used^19^. Introducing a concentration threshold $c_{th}$, the zone of influence of the source node is delimited as the contaminated domain characterized by concentration $c> c_{th}$.

Despite the adopted simplifications, the model provides a satisfactory description of the propagation dynamics in the streets, as discussed in^67^ and shown in Section S1 of the Supplementary Information. This is especially true in the first blocks downwind the source where dynamics in the streets are preponderant with respect to dispersion above roof level and re-entrainment from the external flow is negligible^68^. Moreover, thanks to the conservative hypothesis for propagation in street intersections, the zone of influence delimited by the model includes streets that, although rarely, can be strongly affected by the release. In this sense, the physical simplifications are not restrictive for the estimation of vulnerability, which accounts for local and severe contaminations. On the other hand, the proposed model has an extremely low computational cost and, as reported in the following section, is the starting point for the analytical formulation of a centrality metric for urban vulnerability to airborne releases.

### Centrality metric for the vulnerability of cities

Within the complex network formalism, vulnerability -reported above in terms of extension of the zone of influence of a source node- can be condensed in a centrality metric of the street network. To this aim, the concentration decay along a street from Eq. (3) is considered as a propagation cost, i.e. as the link weight:

4 $$\begin{aligned} \omega _{ij}=e^{\frac{l_{ij}v_{ij}}{u_{ij}h_{ij}}}. \end{aligned}$$

Following a physical-based rationale, we derived in^20^ a tailored centrality metric to identify the most vulnerable areas in a city as the best spreading nodes. The metric is based on the shortest path ($d_{sr}$) between two nodes (*s* and *r*) and is limited by a threshold taking into account the progressive decrease of the contamination potential with distance from the source:

5 $$\begin{aligned} V_{s}=\sum _{r \in \mathcal {V}} \mathcal {H}\left[ \prod _{(i,j)\in D}\frac{1}{\omega _{ij}}-\frac{c_{th}}{c_0}\right] \frac{1}{d_{sr}}, \end{aligned}$$

where $V_s$ is the centrality of node *s*, $\mathcal {V}$ is the set of nodes reachable from *s*, $\mathcal {H}$ is the Heaviside step function, *D* is the shortest path between *s* and $r \in \mathcal {V}$, $c_{th}$ is the threshold concentration for relevant pollution. The length of the shortest path ($d_{sr}$) is defined as:

6 $$\begin{aligned} d_{sr}=|\mathcal {D}|=\min _{\mathcal {P}} \left[ \sum _{(i,j)\in \mathcal {P}} \omega _{ij} : \mathcal {P} \text{ is } \text{ path } \text{ from } \text{ s } \text{ to } \text{ r }\right] . \end{aligned}$$

In^20^, we demonstrated that the centrality of a node (Eq. 5) is very well correlated to the extension of the contaminated area from the source (i.e. the zone of influence of the node). Thus, the higher the centrality of a node, the higher its spreading potential and thus its vulnerability. For $c_0/c_{th}=10$, R-squared between centrality and extension of the zone of influence is 0.94, 0.82, 0.94 and 0.92 for Lyon, Firenze, Manhattan and Paris, respectively.

As mentioned in the Introduction, the number of people exposed to the contamination is not considered in this metric. However, this information could be easily integrated into the model, by modifying Eq. (5):

7 $$\begin{aligned} I_{s}=\sum _{r \in \mathcal {V}} \mathcal {H} \left[ \prod _{(i,j)\in D}\frac{1}{w_{ij}}-\frac{c_{th}}{c_0}\right] \frac{P_{sr}}{d_{sr}}, \end{aligned}$$

where $P_{sr}$ is the number of inhabitants along the shortest path $\mathcal {D}$. Since $d_{sr}$ is defined in Eq. (6) as the overall propagation cost along the path, we can interpret the new metric as a measure for the number of affected people weighted by the concentration decay, i.e. the greater the ventilation of pollutants along the path, the higher $d_{sr}$, the less the impact ($I_s$) of the contamination on the exposed inhabitants $P_{sr}$.

### Topological descriptors

**Figure 6:**
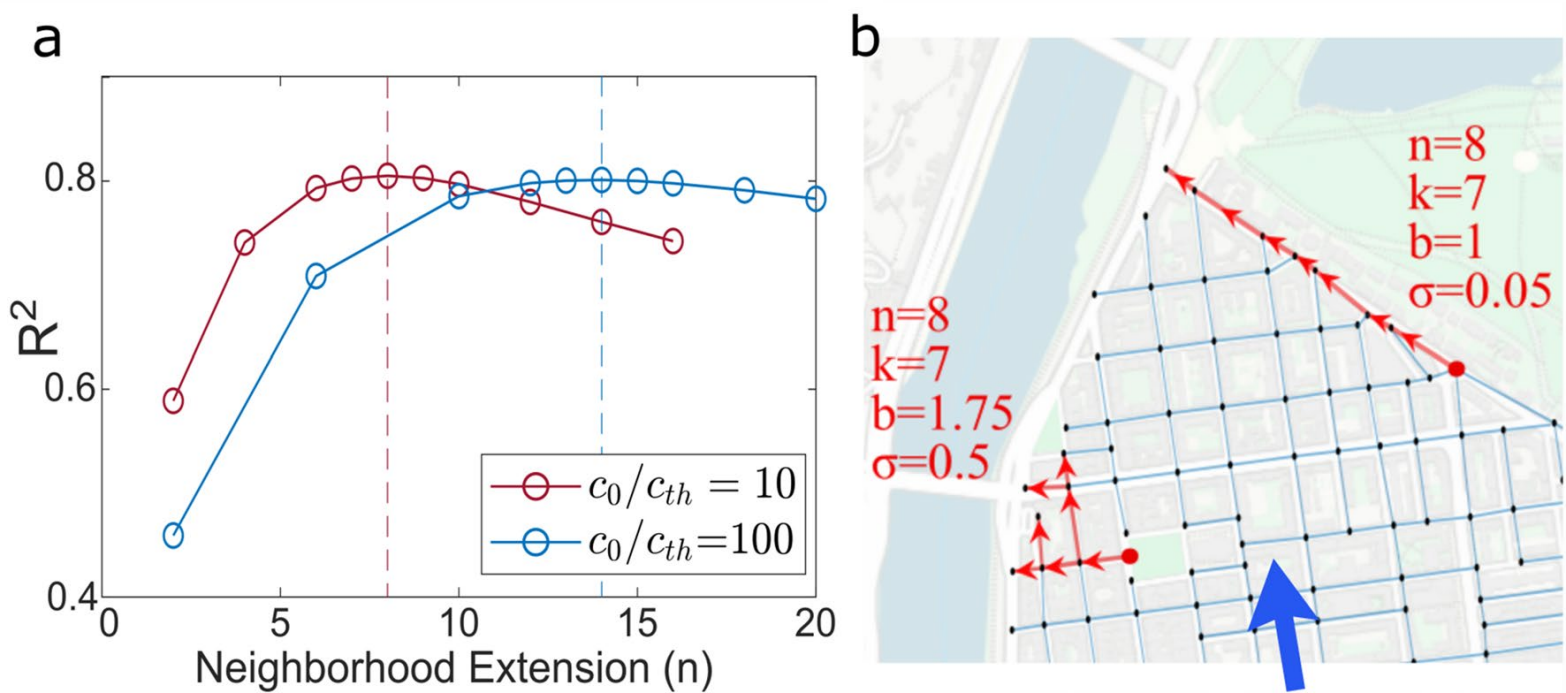
Topological indicators. **(a)** Coefficient of determination for the regression model as a function of the extension of the node neighborhood for two concentration scenario: $c_0/c_{th}=10$ and $c_0/c_{th}=100$. **(b)** Estimate of the topological parameters (*k*, *b*, $\sigma$) for two different nodes of the street network in Lyon, in the same wind scenario (the orientation of the wind is reported as a blue arrow). Background images made with QGIS 2.18 (https://qgis.org).

The topological and geometrical characteristics of the neighborhood of a node proved to be the key indicators for its vulnerability. The neighborhood is delimited as the set of links that can be traveled within a *n-hop* path from the node, where *n* is the value that optimizes the correlation in the regression model in Eq. (1). For the analysis presented in the main text, the neighborhood properties are suitable to predict the spreading potential of the node when $n=8$, i.e. when the neighborhood is extended up to an $8-hop$ topological distance from the node. Evidently, the extension of the neighborhood, *n*, depends on the initial concentration at the source node ($c_0$) and on the threshold concentration for contamination to be relevant ($c_{th}$). As the ratio $c_0/c_{th}$ increases, the phenomenon involves a larger area. Accordingly, the optimal distance for the delimitation of the node neighborhood increases as well (Fig. 6a).

The *n-hop* distance is therefore an additional topological parameter that takes into account the size of the toxic release. Since in this work the release scenario is fixed, this parameter keeps constant and is absorbed in the other predictors.

The other relevant topological indicators in Eq. (1) are the number of links in the neighborhood (*k*), the branching index (*b*) and the standard deviation of street orientation ($\sigma$) with respect to the incident wind.

The branching index assesses whether the neighborhood structure is more linear or more branched (see the two examples of neighborhood in Fig. 6b). It is defined as the average outdegree for the internal nodes belonging to the neighborhood:

8 $$\begin{aligned} b=\frac{1}{m} \sum _{i\in \mathcal {N}}^n \sum _{j\in \mathcal {M}}^m A_{ij}, \end{aligned}$$

where $A_{ij}$ is the adjacent matrix for the neighborhood subnetwork. This matrix describes the connectivity of the links in the subnetwork: the element $A_{ij}$ is equal to 1 if a directed link from node *i* to node *j* exists, is equal to 0 otherwise. $\mathcal {N}$ is the set of the internal nodes in the neighborhood, $\mathcal {M}$ is the set of the neighbors for the $i-th$ node in $\mathcal {N}$, *n* and *m* are the sizes of $\mathcal {N}$ and $\mathcal {M}$, respectively. Note that the information embedded in *b* is different from that embedded in *k*. To enlighten this aspect, we show in Fig. 6b the case of two neighborhoods with same *k* but different *b* and $\sigma$. The neighborhood at the top is composed of 7 links ($k=7$) in succession and with almost identical orientation. Its branching index (*b*) is therefore equal to 1 while the standard deviation of street orientation ($\sigma$) is very low. The lower right neighborhood has the same number of links ($k=7$) but it is characterized by a more branched structure and greater variation in the orientation of the streets. Consequently, the values of *b* and $\sigma$ are considerably higher. In terms of vulnerability, *k* indicates the number of links potentially reachable by the toxic substance released in the source. For a fixed value of *k*, a higher branching index (*b*) reveals that the neighborhood links are topologically closer to the source and therefore can be affected by higher pollutant concentrations. Finally, with a same *k* and *b*, a low $\sigma$ indicates a low variability in the orientation of the streets with respect to the direction of the wind. Consequently, all propagation paths in the neighborhood are effectively activated. In the case of high $\sigma$, some streets, despite being in the downwind neighborhood of the source, are characterized by a longitudinal wind intensity so low as to interrupt the propagation path.

## Supplementary Information

Supplementary Information.
