# Supplementary material for: Vulnerability of cities to toxic airborne releases is written in their topology
Source: Sci Rep. 2021 Nov 29;11:23029. doi: 10.1038/s41598-021-02403-y (PMC8630004; doi:10.1038/s41598-021-02403-y)
Supplement: Supplementary file 1 — Supplementary Information. [file 41598_2021_2403_MOESM1_ESM.pdf]

# Supplementary Information to “Vulnerability of cities to toxic airborne releases is written in their topology”

**Sofia Fellini[1,2*], Pietro Salizzoni[1], and Luca Ridolfi[2]**

[1]Laboratoire de Mécanique des Fluides et d’Acoustique, UMR CNRS 5509, Université de Lyon, École Centrale de Lyon, INSA Lyon, Université Claude Bernard Lyon I, 69134 Écully, France.
[2]Department of Environmental, Land and Infrastructure Engineering, Politecnico di Torino, 10129 Turin, Italy.
[*]sofia.fellini@polito.it

## S1 Assessment of the complex network-based model for gas propagation in the streets

Fig. S1 compares the outcome of the complex network (CN) model with the results from wind tunnel experiments[1] and SIRANE simulations reported in[2]. The experimental urban canopy is composed by square buildings 0.25 m large and 5 cm high, separated by 5 cm wide streets. Above the canopy, a neutral boundary layer is formed with free-stream velocity 5 m/s, displacement height 5 cm and friction velocity 0.22 m/s. The Reynolds number is $1.5 \times 10^4$, which is sufficiently high to ensure a Reynolds-independent flow and therefore a concentration field invariant for different values of the external free-stream velocity[1]. Two different incident angles of the external wind are considered: 152.5° and 170° clockwise angle with respect to the vertical (downward) axis of the network. From these simple input data, we evaluate $u$ and $v$ in each street adopting the parameterizations proposed by[3] and[4], and we compute the concentration profiles along the propagation paths by means of the model reported in the Methods (Eq. 3). In the near field, the experimental concentrations (black diamonds) are in good agreement with the exponential decay given by Eq. 3 (red diamonds). Discrepancies are instead found far from the source, where the effect of re-entrainment from the external plume towards the streets is responsible for the increase of pollutant concentrations along the direction of the external wind. These concentrations are however very low ($< 1\%$) with respect to the concentration near the source. While this effect is well reproduced by SIRANE (blue lines), as it couples dispersion dynamics below and above roof level, it is neglected by the proposed CN model.

This comparison evidences that the adopted CN model is valuable in predicting pollutant dispersion in the first blocks downwind the source where dynamics in the streets are preponderant with respect to dispersion above roof level. Moreover, in the near field, concentrations are slightly overestimated along the lateral (with respect to the wind direction) segments of the network, as the model cautiously assigns the same probability to all the propagation paths downwind each intersection. Under this assumption, the statistically average concentration in the streets loses accuracy but the predicted zone of influence includes streets that can be significantly affected by the release in rare events. In this sense, the limits of the model are not restrictive for the estimation of vulnerability which accounts for local and severe contamination.

To confirm these findings, we conducted a systematical comparison between the outcomes of the CN model and those of SIRANE. Thanks to this software we could perform numerous simulations and thus validate the results of the CN model for all the cities analyzed in this work and for the different wind directions. SIRANE simulates urban air pollution by reproducing the transport of pollutants within the urban canopy, the dispersion in the atmospheric boundary layer above roof level, and the physicochemical processes involved in the transformation of pollutants. For this accurate elaboration, the software requires as input the meteorological conditions of the site and the physico-chemical properties of the pollutants. To the purpose of validation, we considered the scenario of a gaseous pollutant acting as a passive scalar. Regarding the meteorological conditions, the external flow simulated a typical atmospheric boundary layer in neutral conditions. Results in terms of zone of influence were very similar for different intensities of the external wind.

For a source placed in a node, we formally define the zones of influence delimited by the CN model and by SIRANE as the link sets $\mathscr{Z}_{\mathscr{M}}$ and $\mathscr{Z}_{\mathscr{S}}$, respectively. As an example, Fig. S2 reports the case of a point release (green node) in the urban centers of Firenze (panel a-b) and Lyon (panel c-d). The zone of influence (red links) is delimited for $c_0/c_{th} = 10$. Results are very similar, with $\mathscr{Z}_{\mathscr{M}}$ (panel b-d) slightly overestimating $\mathscr{Z}_{\mathscr{S}}$ (panel a-c), as expected.
The interplay between sets $\mathscr{Z}_{\mathscr{M}}$ and $\mathscr{Z}_{\mathscr{S}}$ is described in Fig. S3.a. The overlap $\mathscr{G}$ between the two sets includes the streets correctly captured by the CN model. The set $\varepsilon$ contains the extra streets included in $\mathscr{Z}_{\mathscr{M}}$ that do not belong to the zone of influence $\mathscr{Z}_{\mathscr{S}}$, defined by SIRANE. Finally, $\mathscr{N}$ contains the streets belonging to $\mathscr{Z}_{\mathscr{S}}$ but neglected by the CN model. From

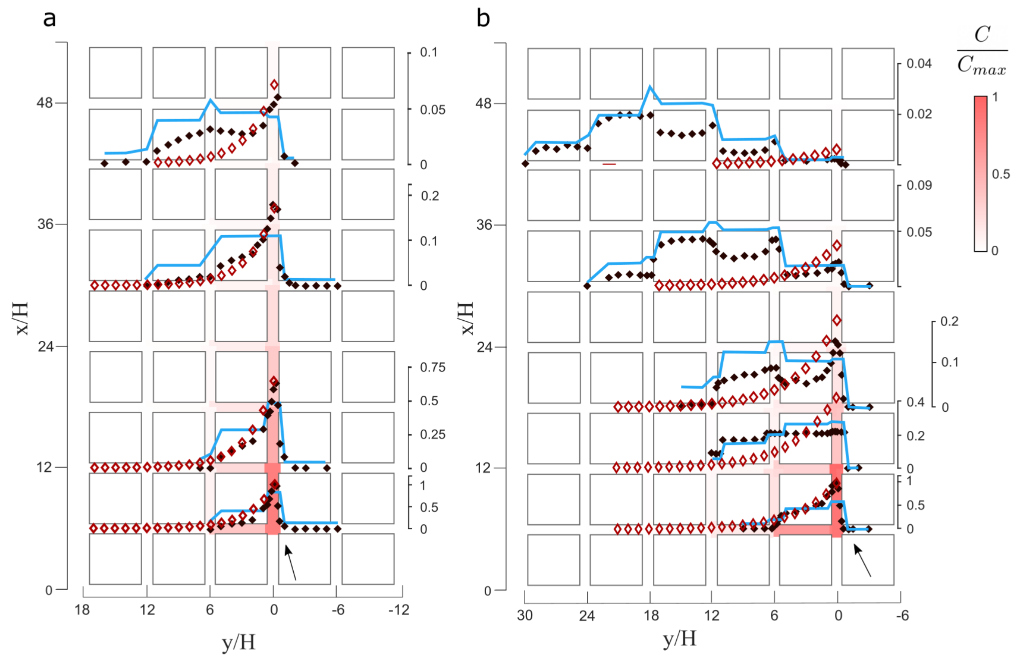


**Figure S1.** Concentration profiles within the streets for a wind orientation of 170° and 152.5° (left and right panels, respectively). Black and red diamonds represent data from wind tunnel experiments and results from the complex network approach, while blue lines report results from the model SIRANE. The background color is the average concentration in the streets estimated from the complex network model. Black vertical axes refer to the normalized concentration, $C/C_{max}$, in the different measurement sections (notice the different scales).

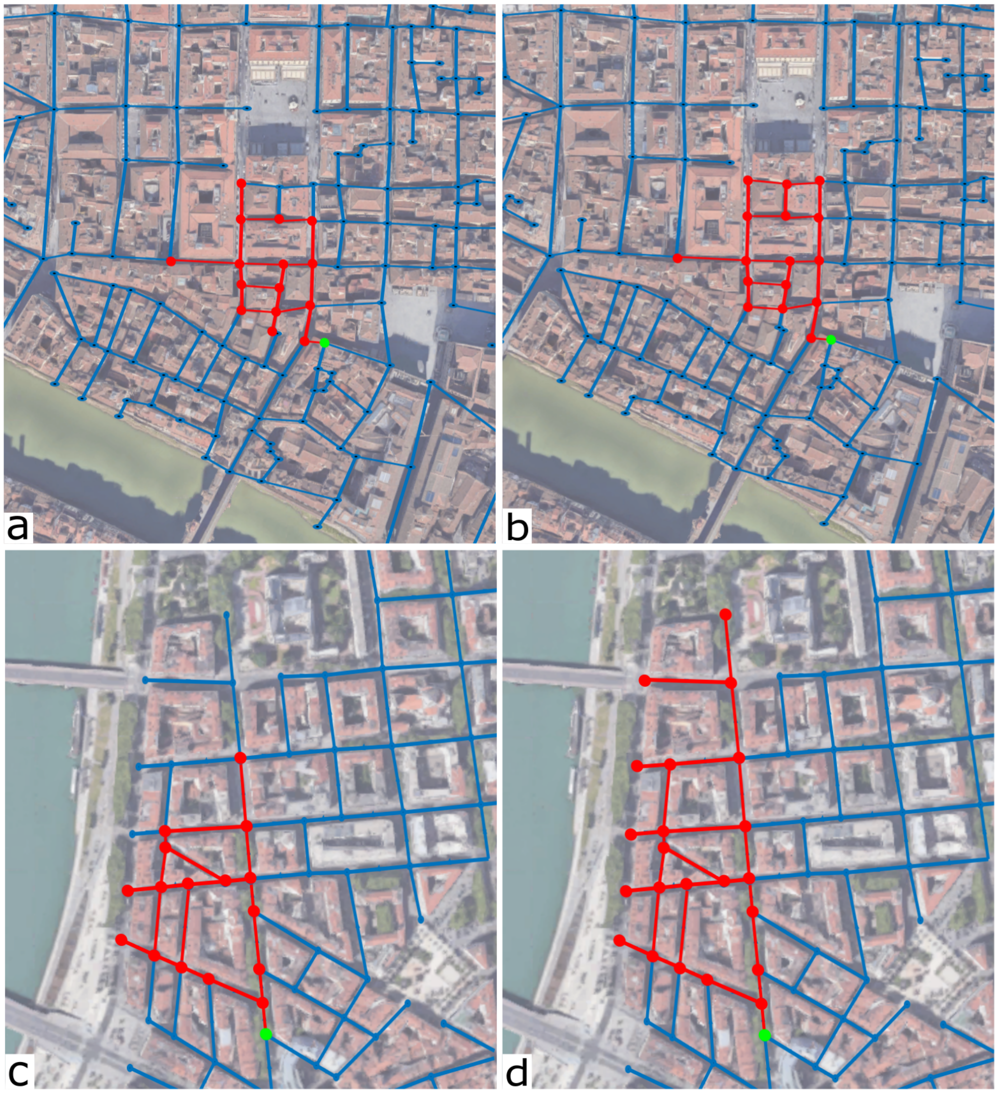


**Figure S2.** Example of zone of influence (red links) for the city of Firenze (a-b) and Lyon (c-d) based on SIRANE simulations and delimited by the proposed network model (left and right panels, respectively). The source node is depicted in green. The wind has an angle of 135° with respect to the main axis of the city. Background images made with QGIS 2.18 (https://qgis.org).

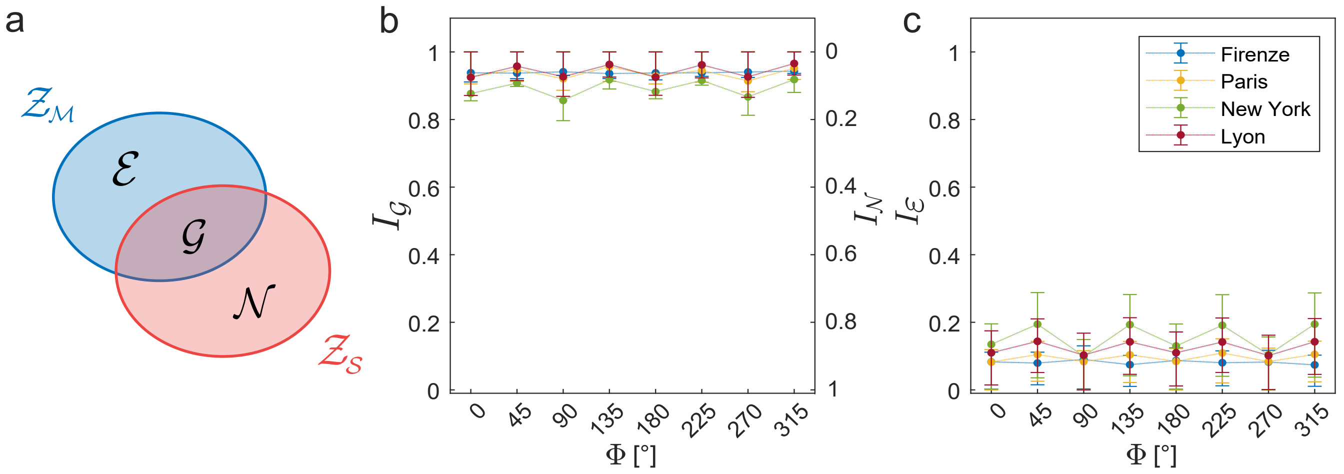


**Figure S3.** a) Relation between the different sets: $\mathscr{Z}_\mathscr{M}$=zone of influence delimited by the network model, $\mathscr{Z}_\mathscr{S}$=zone of influence delimited by SIRANE, $\varepsilon$=extra streets, $\mathscr{N}$=neglected streets. b-c) Value of the three indexes $I_\mathscr{G}$, $I_\mathscr{N}$ and $I_\varepsilon$ for the analyzed cities and different wind orientations. Circles represent the average value for each scenario, while the ends of the vertical intervals show the upper and lower quartiles of the distributions.

this schematization, we define three quality indexes: $I_\mathscr{G}$, $I_\varepsilon$ and $I_\mathscr{N}$. The goodness coefficient $I_\mathscr{G}$ estimates the accordance between the two models:

$$I_\mathscr{G} = \frac{\sum_{i\in\mathscr{G}} l_i \frac{c_i}{c_0}}{\sum_{i\in\mathscr{Z}_\mathscr{S}} l_i \frac{c_i}{c_0}}. \tag{1}$$

$I_\mathscr{G}$ is equal to 1 when all the streets in $\mathscr{Z}_\mathscr{S}$ are also in $\mathscr{Z}_\mathscr{M}$. It is equal to 0, when the CN model cannot capture any contaminated street. In the computation of the error, the streets are weighted according to their length and contamination level. In this way, the longer and more contaminated streets have a greater weight in the estimation of the accordance between the two models. Similarly, the error indexes associated to $\varepsilon$ and $\mathscr{N}$ are:

$$I_\varepsilon = \frac{\sum_{i\in\varepsilon} l_i \frac{c_i}{c_0}}{\sum_{i\in\mathscr{Z}_\mathscr{S}} l_i \frac{c_i}{c_0}}, \qquad I_\mathscr{N} = \frac{\sum_{i\in\mathscr{N}} l_i \frac{c_i}{c_0}}{\sum_{i\in\mathscr{Z}_\mathscr{S}} l_i \frac{c_i}{c_0}}. \tag{2}$$

$I_\varepsilon$ is equal to zero when $\varepsilon$ is empty. It is maximum when all the streets captured by $\mathscr{Z}_\mathscr{M}$ are not in the zone of influence delimited by SIRANE ($\mathscr{Z}_\mathscr{S}$), i.e. when all the streets are wrongly detected. $I_\mathscr{N}$ is equal to zero when $\mathscr{N}$ is empty. It is equal to 1 when all the streets in the zone of influence of SIRANE ($\mathscr{Z}_\mathscr{S}$) are also in $\mathscr{N}$, i.e. when all the contaminated streets are not detected by the CN model. Notice that $I_\mathscr{G} = 1 - I_\mathscr{N}$.

In Fig. S3 the three indexes are reported for multiple scenarios. The accordance ($I_\mathscr{G}$) between the results from the two models is rather high for all the scenarios. Consequently the error associated to the neglected streets ($I_\mathscr{N}$) is considerably low. The error associated to the extra streets ($I_\varepsilon$) is slightly higher, confirming that the network model tends to overestimate the extension of the contaminated areas, when the focus is on significantly high concentrations ($c_0/c_{th} = 10$).

These verifications (carried out against a model that can be taken as a benchmark for its extensive validation and wide use) confirm that the complex network-based model provides excellent results and can be used for the purposes of our study, focused on the structural properties of a city governing its vulnerability to airborne releases.

## References


1. Garbero, V., Salizzoni, P. & Soulhac, L. Experimental study of pollutant dispersion within a network of streets. *Boundary-layer meteorology* **136**, 457–487 (2010).
2. Salem, N. B., Garbero, V., Salizzoni, P., Lamaison, G. & Soulhac, L. Modelling pollutant dispersion in a street network. *Boundary-Layer Meteorol.* **155**, 157–187 (2015).
3. Soulhac, L., Perkins, R. J. & Salizzoni, P. Flow in a street canyon for any external wind direction. *Boundary-Layer Meteorol.* **126**, 365–388 (2008).
4. Salizzoni, P., Soulhac, L. & Mejean, P. Street canyon ventilation and atmospheric turbulence. *Atmospheric Environ.* **43**, 5056–5067 (2009).
